# Controversies and Advances in Gestational Diabetes—An Update in the Era of Continuous Glucose Monitoring

**DOI:** 10.3390/jcm7020011

**Published:** 2018-01-25

**Authors:** Marina P. Carreiro, Anelise I. Nogueira, Antonio Ribeiro-Oliveira

**Affiliations:** Laboratory of Endocrinology, Federal University of Minas Gerais, Belo Horizonte 30130-100, Brazil; marina.carreiro@uol.com.br (M.P.C.); aneliseimp@gmail.com (A.I.N.)

**Keywords:** gestational diabetes, diagnosis, inflammation, lipids, birth weight, glucose

## Abstract

Diabetes in pregnancy, both preexisting type 1 or type 2 and gestational diabetes, is a highly prevalent condition, which has a great impact on maternal and fetal health, with short and long-term implications. Gestational Diabetes Mellitus (GDM) is a condition triggered by metabolic adaptation, which occurs during the second half of pregnancy. There is still a lot of controversy about GDM, from classification and diagnosis to treatment. Recently, there have been some advances in the field as well as recommendations from international societies, such as how to distinguish previous diabetes, even if first recognized during pregnancy, and newer diagnostic criteria, based on pregnancy outcomes, instead of maternal risk of future diabetes. These new recommendations will lead to a higher prevalence of GDM, and important issues are yet to be resolved, such as the cost-utility of this increase in diagnoses as well as the determinants for poor outcomes. The aim of this review is to discuss the advances in diagnosis and classification of GDM, as well as their implications in the field, the issue of hyperglycemia in early pregnancy and the role of hemoglobin A1c (HbA1c) during pregnancy. We have looked into the determinants of the poor outcomes predicted by the diagnosis by way of oral glucose tolerance tests, highlighting the relevance of continuous glucose monitoring tools, as well as other possible pathogenetic factors related to poor pregnancy outcomes.

## 1. Introduction

Our goal is to discuss the most current clinical data on gestational diabetes. A PubMed search up to July 2017 was conducted using the search term “gestational diabetes”, and over 10,000 articles were retrieved. All English language abstracts were read and vetted for relevance to this study, and priority was given to the articles with high quality experimental design and data analysis, especially those published in prestigious journals and most cited. During the review process of the manuscript a novel reference from the second semester of 2017 was then included.

Gestational Diabetes Mellitus (GDM) is a growing public health concern. There are many controversies yet to be clarified, regarding its definition, classification, diagnosis, and outcome determinants. Hyperglycemia in pregnancy is considered a high-risk condition for both the mother and the fetus, and there has been an increasing prevalence of both gestational and preexisting diabetes mellitus (DM) [1]. Increasing global rates of type 2 diabetes are now extending into pregnancy, showing similar poor outcomes to those of type 1 diabetes in pregnancy, particularly when it is associated with other comorbidities [2]. Furthermore, excess maternal weight is adding to the complexity of the condition’s management [2]. Hyperglycemia, caused by GDM or preexisting diabetes, has been associated with adverse pregnancy outcomes, such as macrosomia (birth weight greater than 4000 g), large for gestational age (birth weight above the 90th percentile based on gender and gestational age), shoulder dystocia, cesarean delivery, stillbirth, as well as neonatal complications, including hypoglycemia, hypocalcemia, hyperbilirubinemia, polycythemia and respiratory distress syndrome [3]. Macrosomia is indeed considered a major phenotypic manifestation of the exposition to a metabolic adverse intrauterine environment [4].

Long-term metabolic and cardiovascular implications of hyperglycemia during pregnancy for both the mother and the child are now recognized as having major implications for public health [5]. It has also been demonstrated that in utero exposure to either nutrient excess or deprivation affects fetal metabolic programming, resulting in an increased long-term risk of obesity, DM, and cardiovascular diseases during adult life [2]. These are all compelling reasons to adequately treat diabetic pregnancies and disrupt the intergeneration contribution to ongoing obesity and DM epidemics.

In this manuscript, we discuss controversial issues of great interest such as the International Association of Diabetes and Pregnancy Study Group (IADPSG) diagnostic criteria, hyperglycemia less than overt diabetes in early pregnancy, the role of glycated hemoglobin A1c (HbA1c) in GDM, outcome determinants, as well as treatment target considerations.

## 2. Classification and Diagnosis

### 2.1. Controversies on the IADPSG Diagnostic Criteria

There has been great effort to standardize the diagnostic criteria of GDM, although controversies remain and there is not a single universally accepted recommendation. The O’Sullivan and Carpenter and Coustan diagnostic criteria (Table 1) were established based on the maternal risk of developing diabetes after pregnancy, and not necessarily on identifying pregnancies with increased risks of adverse perinatal outcomes. Thereafter, the landmark Hyperglycemia and Adverse Pregnancy Outcomes (HAPO) study was designed to clarify the relationship between glucose levels lower than those diagnostic of diabetes at 75-g Oral Glucose Tolerance Test (OGTT) and perinatal outcomes [3].

The IADPSG [6] has published in 2010 a consensus panel recommendation on the classification and diagnosis of hyperglycemia, mainly based on the HAPO study [3]. The main objective of IADPSG was to foster an international approach in enhancing the quality of care and research in the field of diabetes in pregnancy [6]. It was proposed that GDM was to be diagnosed whenever a single value out of three measurements following a 75-g two-hour OGTT, performed between 24 and 28 weeks of gestation, is: fasting blood glucose (FBG) ≥ 5.1 mmol/L, one-hour value ≥ 10 mmol/L, or two-hour value ≥ 8.5 mmol/L [6].

With regard to GDM classification, the IADPSG has suggested firstly to distinguish women with previous diabetes, thus defining GDM as “hyperglycemia with first recognition during pregnancy that is not overt diabetes [6]” instead of any hyperglycemia first recognized in pregnancy, as it had been previously recommended. This is of great importance insomuch as there are increasing numbers of undiagnosed diabetic women of childbearing age, and they carry a much higher risk of maternal and fetal complications, thus requiring earlier therapeutic intervention. Therefore, if at least one of the following criteria is reached anytime during pregnancy, then the diagnosis should be overt diabetes instead of GDM: FBG ≥ 7 mmol/L, or random glucose ≥ 11.1 mmol/L, or HbA1c ≥ 6.5% [6].

These new diagnostic criteria were endorsed by the American Diabetes Association (ADA), the Endocrine Society, and the World Health Association (WHO) [1,7,8]. Of note, these criteria clearly increase the prevalence of GDM, by including a group with less intense metabolic impairment although excluding those with previous diabetes, as compared to the former widely used Carpenter and Coustan two-step approach.

The American College of Obstetricians and Gynecologists (ACOG) otherwise rejected these new recommendations, arguing whether the treatment of this new group of milder GDM patients would really improve pregnancy outcomes [9]. They maintained the two-step recommendation of a one-hour screening test with a 50-g glucose load (step 1) followed by a three-hour 100-g glucose tolerance test for those found to be abnormal on screening (step 2) [10]. The ACOG recommends the interpretation of the three-hour 100-g glucose tolerance according to either Carpenter and Coustan [11] or the National Diabetes Data Group (NDDG) criteria [12] (Table 1), although in their 2017 updated practice bulletin they say that one elevated value, as opposed to two, may be used for the diagnosis of GDM [10]. This new consideration was based on a systematic review that concluded that women with one abnormal value on the three-hour 100-g GTT were at increased risk for adverse pregnancy outcomes [13].

Societies from other countries such as Spain and New Zealand have also not endorsed the IADPSG criteria [5]. It is noteworthy, however, that the ADA [1] considers both the one-step criteria proposed by IADPSG [6], and the two-step method supported by ACOG [10] as acceptable.

The National Institute of Health (NIH) points out that the IADPSG approach, in addition to operational advantages, would potentially allow the standardization of best practices in patient care, and then provide better comparability of research outcomes. However, the expected significant increase in GDM prevalence by using the IADPSG criteria, from 5–6% to 15–20%, has raised a concern from the NIH that the impact of treatment on pregnancy outcomes should be better evaluated, as well as its cost-effectiveness before fully adopting the new criteria proposed by IADPSG [14].

Weile et al. [5] reviewed the cost-effectiveness and cost-utility of different strategies of GDM screening and management by way of a systematic literature search of articles published between 2002 and 2014, in which just two studies included the new IADPSG screening and diagnostic criteria [15,16]. The authors concluded that it would not be possible to define the global cost-effectiveness properly until we have standardized screening, diagnosis, treatment and outcome impacts analyzed through standardized cost measurements.

Interestingly, the IADPSG diagnostic criteria for GDM have been used in China since 2011. In 2014, a large study enrolling over 25,000 Chinese patients with GDM diagnosed by the IADPSG criteria concluded that, despite an increase in GDM prevalence, the treatment of the women identified by these criteria was related to a lower risk of adverse pregnancy outcomes [17].

### 2.2. Controversies in Early Pregnancy and the Role of HbA1c in Pregnancy

However, it is noteworthy that these new criteria were not derived from data during the first half of pregnancy. In regard to hyperglycemia “less than overt diabetes” in early pregnancy, especially before 24 weeks of gestation, there is not enough data to propose a new classification or diagnostic cutoffs [18]. The IADPSG had proposed that FBG in the range of 5.1–6.9 mmol/L in early pregnancy could be enough to be considered a diagnosis of GDM [6]. The WHO had also accepted this recommendation, although they argued the benefit of this early diagnosis and treatment [8]. Recently, this recommendation was re-evaluated by members of the IADPSG council [18], based on two publications that have strongly challenged it, showing that FBG ≥ 5.1 mmol/L in early pregnancy is not a good predictor of GDM diagnosis between 24 and 28 weeks of gestation, although it could be considered as a marker of a high-risk group [19,20]. Indeed, the women with hyperglycemia less than DM in early pregnancy do not have an adequate label and, although often classified as GDM, they probably carry some degree of hyperglycemia from outside of pregnancy. It was suggested to use the term “early GDM”, in contrast to “late GDM” for those who are diagnosed in late pregnancy. However, it is important to recognize that these are different clinical entities, and that only “late GDM” is really a consequence of pregnancy [21]. Interestingly, only half of those patients with FBG in the range of 5.1–6.9 mmol/L in early pregnancy fulfill the criteria for late GDM thereafter [22].

Concerning HbA1c, there is no cutoff point to establish the diagnosis of GDM, neither by IADPSG nor by others, although a value ≥ 6.5% is diagnostic of overt diabetes anytime during pregnancy, as outside of pregnancy. Non-pregnant individuals with HbA1c between 5.7% and 6.4% are labelled as “prediabetes” [1], but there is not an equivalent cutoff point during pregnancy. Pregnancy physiological changes often lower HbA1c levels and pregnancy-specific ranges are yet to be established. In later pregnancy and postpartum, OGTT is more useful than HbA1c at detecting glucose derangements [21]. Nevertheless, the potential usefulness of an early HbA1c test has been pointed out [21]. It was demonstrated by a large study from New Zealand that HbA1c ≥ 5.9% is an optimal cutoff for identifying women with preexisting but undiagnosed diabetes in early pregnancy, and that it is also a marker for adverse pregnancy outcomes [23].

It may be worthwhile at least to consider women with FBG ≥ 5.1 mmol/L and/or HbA1c ≥ 5.9% who do not match the criteria for overt diabetes as a high-risk group. These high-risk patients could possibly benefit from earlier advice regarding diet, physical activity and monitoring for excessive weight gain, especially those who have other known risk factors for GDM, such as age, ethnicity, excess weight, history of previous GDM, family history of type 2 diabetes, signs of insulin resistance, among others. However, there is not enough data to ensure that early intervention will necessarily translate into better outcomes, and large randomized controlled trials (RCTs) are required to further address this issue.

## 3. Current Glucose Treatment Targets in Diabetic Pregnancies

It has been recommended to keep blood glucose levels as close to normal as possible in the management of diabetic pregnancies; thus, it is of great interest to understand normal pregnancy glucose profiles in order to establish glucose treatment targets. Hernandez et al. [24] have systematically reviewed and pooled more than 45 years of normal pregnancy glucose data, mainly from studies conducted in the USA and Europe. They showed that normal glucose patterns were very similar across different studies, and glucose levels were generally lower than expected, including FBG of 3.9 ± 0.4 mmol/L, one-hour postprandial glucose of 6.0 ± 0.72 mmol/L, two-hour postprandial glucose of 5.5 ± 0.55 mmol/L, and 24-h mean of 4.9 ± 0.55 mmol/L. Otherwise, Barbour et al. [25] have demonstrated that obese pregnant women with normal glucose tolerance show higher glucose values than non-obese women, even those on strict eucaloric diets. In fact, glucose profiles evaluated by continuous glucose monitoring (CGM) in obese women showed higher fasting, postprandial, nocturnal and mean 24-h glucose values than those in normal-weighted pregnant women in the same gestational period, even with normal glucose tolerance. Interestingly, the strongest observed predictors of infant adiposity were related to maternal body mass index (BMI) and lipids, such as non-esterified fatty acids and triglycerides, while glucose values were of less importance [25].

The current glucose treatment targets recommended by most international organizations in pregnant women with diabetes are: FBG ≤ 5.3 mmol/L, one-hour postprandial (PP) ≤ 7.2–7.8 mmol/L, and two-hour PP ≤ 6.7 mmol/L [1,7,9].

Based on the above-mentioned findings, it was suggested by Hernandez et al. [24] to test lower therapeutic targets of postprandial (PP) 1 h < 6.77 mmol/L and 2 h < 6.11 mmol/L, as opposed to the current recommendations, while keeping the FBG recommendation of 5.11 mmol/L (HAPO) [3]. By targeting these lower glucose values we would closer mimic normal pregnancies and possibly further reduce adverse outcomes, such as macrosomia. Nevertheless, the risks of maternal hypoglycemia related to an increased use of insulin, as well as of small-for-gestational-age neonates, should not be underestimated and should be closely monitored, as a relationship between the level of glycemic control and neonatal weight has been shown [26]. Intriguingly, literature does not differentiate glucose target recommendations between GDM and type 1 or type 2 diabetes during pregnancy.

Although there is an agreement concerning glucose treatment targets, they were not established based on strong evidence. Historically, therapeutic targets were mainly based on diagnostic thresholds and normal pregnancy glucose values. An ideal study to clarify optimal glucose targets in pregnancies affected by diabetes would compare the impacts of different glucose targets on outcomes. However, such a study is yet to be done.

Prutsky et al. [27] conducted a systematic review and meta-analysis to establish the optimal glucose targets in pregnant women with type 1, type 2, and GDM. They concluded that, although the literature is limited by the paucity and heterogeneity of data, there is evidence that FBG < 5 mmol/L prevents macrosomia as well as other adverse outcomes, such as preeclampsia and neonatal hypoglycemia in women with GDM. Otherwise, for women with diabetes before pregnancy, the data are not conclusive. They suggest that RCTs comparing different glucose targets and their impact on pregnancy outcomes should be performed.

## 4. Comprehensive Glucose Management: Beyond Glucose Time Point Measures

Glucose control is obviously a key point in GDM treatment and it certainly impacts favorably on reducing adverse outcomes such as macrosomia. However, it still does not normalize outcomes, even when, according to current guidelines [28,29], excellent glucose control is achieved. Indeed, current glucose targets might be either too high or not sensitive enough to detect important normal daily glucose variations affecting overgrowth. Studies with CGM have helped to unmask important features such as glucose exposure and variability, in addition to glucose peaks related to meals [30,31,32]. They have also been extremely useful in strengthening our understanding of normal pregnancy glucose profiles, as well as to disclose differences from diabetic pregnancies [33]. The relationship between CGM data and pregnancy outcomes needs further clarification, in order to better understand which glucose features really impact on outcomes. Indeed, important features of glucose profiles, such as glucose variability and others, have not been properly targeted in the current therapeutic recommendations, although current investments in different monitoring schedules, such as self-monitoring of blood glucose by fingerstick glucose determinations at different times of the day, as well as continuous glucose monitoring, have been made [34].

Different glucose monitoring schedules and techniques have not changed pregnancy outcomes in women with pre-existing diabetes, as evidenced in a recent Cochrane review [34]. One possible reason could be that therapeutic strategies do not take into account all available glucose data information. Otherwise, it has been demonstrated that CGM during pregnancy impacts favorably on pregnancy outcomes when used by women with GDM in combination with glucose variability parameters, as well as glucose time points as treatment determinants [35]. This reinforces the importance of using all available information obtained through glucose monitoring in a targeted therapeutic approach.

CGM is a potentially useful tool in clarifying which glucose parameters are best related to poor outcomes. However, the analysis and interpretation of the enormous amount of complex data provided by CGM is challenging. There have been some publications with suggestions on how to interpret these data, including the identification of some parameters which may be of specific interest during pregnancy. Hernandez and Barbour [36] have proposed a standard approach to CGM data in the study of fetal growth and infant outcomes. They propose the analysis of specific time points related to meals, as well as the time spent in predefined ranges, glucose level means during periods of the day and night, and the 24-h area under the curve of measurements. Mazze et al. [30] suggest means of measuring glucose exposure and variability in pregnancy, and they use summary measures and a program called Ambulatory Glucose Profile (AGP). Law et al. [37] suggest the use of Functional Data Analysis (FDA), a technique used to address temporal trend analyses.

Law et al. [37] conducted a very interesting study addressing the correlation of CGM data and large for gestational age (LGA) occurrence in order to understand the role of residual glucose variation in clinically well-controlled pregnancies. The authors identified distinct temporal patterns of glucose, and specific times of the day that maternal glucose excursions were associated with LGA. The FDA technique showed that short-term differences in glucose levels underlie significant differences in the summary statistical indices of average glucose levels and glucose variability across each trimester [37].

Furthermore, there is evidence that the glucose parameters related to glucose variability, and not those related to specific time points, correlate with fetal outcomes. It has been demonstrated that glucose level excursions above 7.2 mmol/L, which can occur at different times of the day, correlate with fetal growth better than fingerstick glucose time point measurements [31]. In a recent publication, McGrath et al. suggested that glucose variability, in addition to HbA1c, should be targeted in order to reduce macrosomia in type 1 diabetic pregnancies [38].

**Figure 1:**
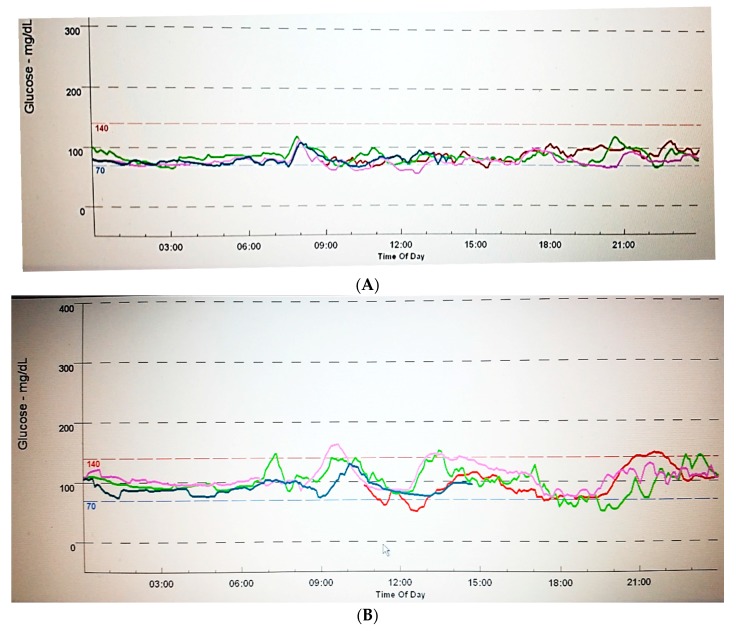
Seventy-two-hour Continuous Glucose Monitoring Data (mg/dL) taken from the CGMS monitor (1 mg/dL = 0.05 mmol/L). Each line represents a 24-h monitoring. (**A**) Non-diabetic pregnant woman; (**B**) Gestational diabetes woman before treatment.

Corroborating these findings, we have recently published a study designed to compare the glucose profiles of GDM patients, diagnosed by using IADPSG criteria, with those of normal pregnancies (NDM), and then to address the role of dietary counseling. The GDM patients were submitted to CGM either before (GDM1) or after dietary counseling (GDM2), and were then compared to healthy controls. Figure 1 shows examples of the CGM results. Of note, during the 72-h monitoring period, all the women registered their food intake, through which it was ensured that those from the groups NDM and GDM1 had similar and higher amounts of simple carbohydrates than those from the GDM2 group, who showed compliance to dietary counseling. Most women (81.8%) in the GDM groups had fasting blood glucose levels less than 5.3 mmol/L, suggesting mild GDM. Moreover, they were non-obese, and those who needed insulin therapy were excluded. We showed that the main observed differences, which were found in comparisons between NDM and GDM1 groups, were related to higher glucose variability and increased number of excursions above 7.8 mmol/L, while glucose exposure was somewhat similar among the groups. There were also differences in glucose time points, and postprandial values were higher at breakfast and dinner times in the diabetic group before dietary counseling. Nevertheless, all values were below current therapeutic targets [1,7,9]. On the other hand, the group studied after two weeks of dietary counseling was able to keep glucose values similar to those observed in normal pregnancies. This study suggests that targeting specific glucose time points may occasionally miss important glucose variations. It also suggests that, whenever glucose exposures are equal, variability and/or other factors other than glucose may be related to poor outcomes, such as fetal overgrowth [33].

Glucose variability, as accessed by way of CGM, has already been linked to poor pregnancy outcomes and it has been shown to be at least partly independent from chronic hyperglycemia [39]. Glucose fluctuation triggers endothelial dysfunction along with the activation of pathological pathways which leads to tissue damage [40].

Interestingly, women with previous GDM, even with normal OGTT one year postpartum, still have a higher glucose variability, suggesting that their glucose metabolism impairment is not normalized after the end of pregnancy, although not detectable by postpartum OGTT [41]. This higher glucose variability could be the link between previous gestational diabetes and endothelial dysfunction, and, since it lasts beyond pregnancy, it could explain why previous gestational diabetes is independently associated with increased carotid intima-media thickness, in a similar way to metabolic syndrome [42]. Recently published data suggest that women with a previous pregnancy complicated by mild GDM are indeed at high risk of developing metabolic syndrome in 5–10 years, although subsequent pregnancies do not further increase this risk [43].

Furthermore, studies conducted on groups at high diabetes risk, such as having mild or subclinical glucose metabolism disorders, have also shown differences in CGM glucose parameters, suggesting that glucose variability and levels above predefined thresholds seem to be the earliest detectable glucose abnormalities within the purview of carbohydrate metabolism disorders [44,45]. Glucose variability could, therefore, be associated with the pathogenetic mechanisms of poor outcomes in pregnancies, via oxidative stress and endothelial dysfunction, even with mild glucose metabolism impairment. It could also be an indicator of insulin resistance and other metabolic derangements such as lipid and inflammatory disorders which are themselves also linked to fetal overgrowth [46,47].

It is noteworthy that glucose variability has been better analyzed by continuous glucose monitoring, accompanying a considerable evolution in monitoring devices. The new technology called Flash Glucose Monitoring (FGM) does not require fingerstick calibration, and its results can be used to support clinical decisions in most situations. Moreover, the FreeStyle Libre^®^ flash glucose monitoring (Abbott, Alameda, CA, USA) data is available to be accessed by AGP. Therefore, the availability of glucose profiles through these new technologies will enhance the feasibility of using the results of glucose variability and excursions in clinical practice [48].

## 5. Comprehensive Pregnancy Management beyond Glucose

Nutrients and metabolites other than glucose certainly play a role in fetal growth and fat accretion [49]. Maternal obesity is an independent risk factor for macrosomia, even without diabetes, and there are more LGA babies born to overweight and obese mothers than those born to diabetic mothers [50]. Common features between obesity and diabetes such as inflammation, insulin resistance, and lipids are known to interfere with fetal growth [4].

The role of inflammation was first demonstrated in 1993 [51] by an observation that the adipose tissue produces pro- and anti-inflammatory mediators (adipokines), which are associated with BMI and insulin resistance [52]. It has also been shown that inflammation predicts the development of type 2 diabetes, which itself is considered to be an inflammatory condition [53]. There is also evidence that inflammation contributes to insulin resistance as well as glucose intolerance during pregnancy [54]. Furthermore, non-diabetic women with previous GDM have a pro-inflammatory profile of adipokines [55]. A possible contributing factor to inflammation in obesity is the chronic excess of macronutrient intake, and it has been demonstrated that dietary restriction in obesity leads to a significant reduction in oxidative stress [56]. Recently, gut microbiota, which are also influenced by diet, have been linked to obesity, inflammation and insulin resistance [57]. Interestingly, overweight and lean pregnant women present distinct compositions of gut microbiota [58]. Furthermore, maternal obesity is related to increased meta-inflammation in the placenta, as well as in maternal adipose tissue and plasma, which could possibly influence placental permeability and the fetal influx of energy substrate, such as lipids and amino acids, thus affecting fetal growth and fat accretion [4,28,59].

During pregnancy there is an increase and redistribution of maternal adipose tissue with changes in the adipokines production. Adiponectin, which has antidiabetic properties, usually decreases from mid-pregnancy. In contrast, leptin, which is related to body weight and insulin resistance, increases throughout pregnancy. Recently, it has been demonstrated that adiponectin decreases mostly in mothers giving birth to LGA infants. It has been suggested that low levels of adiponectin could independently contribute to the development of LGA infants due to its influence in placenta permeability. Otherwise, leptin levels were not shown to be related to infant birthweight [60].

Another interesting study, performed on a subset of participants of the HAPO, was designed to examine the associations between inflammatory mediators and maternal glucose levels and neonatal size [46]. They found that inflammatory mediators are associated with glucose during pregnancy in women without overt diabetes. They also found a negative association of maternal adiponectin levels with birthweight. Interestingly, although C-reactive protein (CRP) and plasminogen activator inhibitor-1 (PAI-1) have both been described as being associated with insulin resistance and diabetes, a negative association of these with birthweight has been found. Further studies are needed to clarify the mechanisms behind these associations.

There is also compelling evidence that lipid abnormalities, especially triglycerides, are associated with adverse pregnancy outcomes, including macrosomia, both in women with normal glucose metabolism and in those with overt or gestational diabetes [61]. An interesting study showed that a pre-pregnancy adverse lipid profile may predict GDM and that small dense LDL particles lead to oxidative stress, which may compromise both insulin secretion and action [62]. Moreover, it was also demonstrated that early second trimester lipid biomarkers correlate with later OGTT, possibly predicting GDM [63]. Importantly, maternal serum triglycerides have been demonstrated to be the strongest predictors of fetal overgrowth both in normal and GDM pregnancies [47], while increased free fatty acids and triglycerides are also recognized features of insulin resistance [45]. Furthermore it is also possible that dietary fat influences cytokine expression, inflammation, and insulin resistance [59].

Interestingly, our group showed a change in the circulating profile of the renin-angiotensin system components for gestational diabetic women. Increases in angiotensin I and angiotensin-(1-7) in normal pregnancies have been demonstrated. In contrast, angiotensin-(1-7), which has vasodilator properties, has not shown an increase in cases of gestational diabetes. This imbalance could be associated with insulin resistance and endothelial dysfunction [64].

The mother’s body weight certainly also impacts on fetal growth and pregnancy outcomes. HAPO data was secondarily analyzed in order to examine the relative contributions of GDM and obesity to adverse maternal and neonatal outcomes [50]. GDM was defined by IADPSG criteria [6] and obesity was defined as BMI ≥ 33.0 kg/m^2^ at 24–32 weeks gestation. Data from 23,316 women were available for analysis, and 3198 (13.7%) of them were obese and 3746 (16.1%) had GDM, of whom 25% were also obese. It was demonstrated that both GDM and obesity are independently associated with adverse pregnancy outcomes, including macrosomia, and, when concurrent, the risks are substantially higher than with either GDM or obesity alone [50]. Also, the risk of macrosomia in women with well-controlled GDM is related to pre-pregnant BMI. Langer et al. [65] have shown that overweight women with well-controlled GDM on diet al.one had a 50% greater risk of giving birth to a macrosomic neonate as compared to women of normal weight with GDM. Excessive weight gain during pregnancy also increases the risk of macrosomia, as indicated by recently published reviews [66]. Interestingly, women with well-controlled GDM, by way of insulin, had no increased risk of macrosomia [65]. This protective effect of insulin has been demonstrated in other studies and it could be due to its effect on lipid metabolism as well as its anti-inflammatory actions, beyond its obvious effect on glucose metabolism [67].

There are some recognized features that predict glucose impairment during and outside of pregnancy. Ferraninni et al. [68] has shown that subjects who develop glucose derangements and DM share common phenotypic features such as higher prevalence of familial diabetes, older age, and higher waist-to-hip ratio, years before clinical diagnosis [68]. Moreover, lipid abnormalities and a pro-inflammatory profile predict GDM, and women with previous GDM maintain the features of metabolic syndrome even years after pregnancy [42,53,62]. It seems that these women have a predisposition to glucose derangements, which is exacerbated by pregnancy, when hyperglycemia becomes clinically evident as well as other imbalances detected in lipids and inflammatory mediators. All these metabolic alterations, as well as maternal obesity and weight gain during pregnancy, seem to affect fetal growth. Therefore, efforts should be made to target them all, ideally before conception, in order to normalize the intra-utero environment and then prevent fetal overgrowth, thus disrupting the intergeneration transmission of continued obesity as well as diabetes epidemics.

Fetal growth may also be influenced by epigenetic mechanisms, even before intrauterine environment exposure. It is accepted that maternal prenatal nutrition impacts on fetal development. In a recent review, Dunford and Sangster [69] found some evidence suggesting that maternal, and even paternal nutrition, around the time of conception, may impact the lifelong risk of metabolic syndrome in offspring, through epigenetic imprinting.

## 6. Conclusions

There has been much recent research on diabetes and pregnancy. Although the HAPO study resulted in the IADPSG diagnostic criteria recommendation, these are still not accepted by all societies and the cost-effectiveness of its use is yet to be determined. Moreover, the correlation of OGTT glucose time points with adverse pregnancy outcomes was shown, although we still need further research into the real determinants of adverse pregnancy outcomes. Importantly, we are not currently able to normalize diabetic pregnancy outcomes even when achieving current glucose therapeutic targets or by using CGM tools throughout pregnancy. Thus, it is clear that we need to advance our understanding of what we regard as GDM, expanding our knowledge to include other nutrients and inflammatory factors, which are also likely implicated during fetal development.

## Figures and Tables

**Table 1:** ACOG diagnostic criteria for the 100-g OGTT to diagnose GDM (Step 2).

	Plasma or Serum Level Carpenter and Coustan	Plasma Level NDDG
Fasting blood glucose	≥5.3 mmol/L (95 mg/dL)	≥5.8 mmol/L (105 mg/dL)
One hour	≥10.0 mmol/L (180 mg/dL)	≥10.6 mmol/L (190 mg/dL)
Two hours	≥8.6 mmol/L (155 mg/dL)	≥9.2 mmol/L (165 mg/dL)
Three hours	≥7.8 mmol/L (140 mg/dL)	≥8.0 mmol/L (145 mg/dL)

The 100-g OGTT is performed when glucose level one hour after 50 g glucose load test, at 24 to 28 weeks, meets or exceeds 7.5 or 7.8 mmol/L (135 or 140 mg/dL) (Step 1). The diagnosis of gestational diabetes is made at 24 to 28 weeks of gestation when at least two glucose levels meet or exceed the above levels, either Carpenter and Coustan or NDDG values (Step 2). ACOG: American College of Obstetricians and Gynecologists; OGTT: Oral glucose tolerance test; GDM: Gestational Diabetes; NDDG: National Diabetes Data Group.
